# A Brief Review on the Resistance-in-Series Model in Membrane Bioreactors (MBRs)

**DOI:** 10.3390/membranes9020024

**Published:** 2019-02-01

**Authors:** Gaetano Di Bella, Daniele Di Trapani

**Affiliations:** 1 Facoltà di Ingegneria e Architettura, Università degli Studi di Enna “Kore”, Cittadella universitaria, 94100 Enna, Italy; 2 Dipartimento di Ingegneria, Università degli Studi di Palermo, Viale delle Scienze, 90128 Palermo, Italy; daniele.ditrapani@unipa.it

**Keywords:** cake deposition, reversible and irreversible fouling, MBR, physical cleaning, pore blocking, RIS model

## Abstract

The cake layer deposited on the membrane modules of membrane bioreactors (MBRs), especially under a submerged configuration, represents a relevant and fundamental mechanism deeply influencing the development of membrane fouling. It negatively affects the total resistance to filtration, while exerting a positive effect as a “pre-filter” promoting the “dynamic membrane” that protects the physical membrane from internal fouling. These two opposite phenomena should be properly managed, where the submerged membranes are usually subjected to a periodical cake layer removal through ordinary (permeate backwashing and air scouring) and/or irregular cleaning actions (manual physical cleaning). In this context, the physical removal of the cake layer is needed to maintain the design filtration characteristics. Nevertheless, the proper evaluation of the effect of physical cleaning operations is still contradictory and under discussion, referring in particular to the correct evaluation of fouling mechanisms. The aim of the present work was to summarize the different aspects that influence the fouling investigations, based on simple models for the evaluation of the resistance to filtration due to the cake layer, through physical cleaning operations.

## 1. Introduction: General Overview

The membrane bioreactor (MBR) process represents a well-established technology and a valuable alternative to conventional activated sludge (CAS) systems, due to the growing awareness of environmental issues and the need for the sustainable exploitation of water as a scarce resource [1].

However, the spread of this technology is still hindered by membrane fouling, wherein the complete knowledge of fouling mechanisms, their development and operational consequences, is still not entirely clear [2,3,4,5].

Membrane fouling is an extremely complex phenomenon and the evaluation of deposition mechanisms, as well as the properties of foulant agents, is far from being understood [6,7,8].

Owing to the high complexity of mixed liquor composition, it is quite difficult to discriminate the influence of each parameter, physical or biological, towards the fouling development. Amongst the main foulant agents, it is worth considering the dimension of the activated sludge flocs, cellular dispersion, organic and inorganic compounds, biomass activity, and extracellular polymers production [9,10,11].

Over the last years, a large amount of research has been conducted with the aim of improving knowledge of membrane fouling. The reported studies have focused on the effects of every single parameter (in particular bacterial consortium, proteins, and colloidal matter) on the micro-ultrafiltration process [12,13,14,15]. 

The technical literature has also focused on the main technical-management parameters that might be influential. The effects of permeate flux, membrane hydrophobicity, and membrane pore size have been thoroughly investigated [16,17,18,19,20]. Furthermore, fouling complexity is emphasized by the highly unstable nature of some substances, such as natural organic matter (NOM), extracellular polymeric substances (EPSs), and humic substances. Indeed, these substances can exert a significant negative influence on the deposition mechanisms depending on the variation of the operational/management conditions [4,21,22,23]. In particular, the interactions between the colloidal matter and the cake layer deposited on the membrane surface may be influenced by several variables. As a consequence, significant differences may arise in terms of permeability and persistence of the deposited layer, even in treatment plants that are characterized by the same configuration and operational conditions [7,24,25].

Theoretically, membrane fouling relies on two fundamental phenomena—colloids, bacteria, protozoa, and virus’s deposition within the membrane pores (internal fouling) and the superficial deposition of biological flocs and suspended particles with an average size higher than the membrane pores (external deposition) [18,26,27]. Nevertheless, these mechanisms are highly related to the membrane pore size (either microfiltration or ultrafiltration, for instance). Referring in particular to cake layer formation, it mostly relies on flocs/particles deposition on the membrane surface (the latter having an average size higher than the membrane porosity). However, under specific conditions (stress characteristics, liquid bulk features, and activated sludge physiology), colloidal matter can also be incorporated within the cake, establishing the so-called “dynamic membrane”. 

Although fouling mechanisms in MBRs have been thoroughly investigated, many discrepancies have been reported in the technical literature. A different and contradicting role has been assigned by many authors to some parameters, like the suspended solids (SS) concentration, depending on other “stress conditions” operated by secondary factors like pH and temperature [8,28]. As an example, according to Hong et al. [29], the effect of mixed liquor concentration is not relevant in the range 3.6–8.4 gTSS L^−1^. In contrast, according to Le-Clech et al. [30], the TSS concentration of mixed liquor produces a decrease in the permeate flux for values lower than 12 gTSS L^−1^. However, these values are significantly lower than the values (30–40 gTSS L^−1^) originally proposed by Yamamoto et al. [31]. Nagaoka et al. [32] attributed the drastic reduction in permeability during filtration to the formation of a dense cake layer on the membrane surface, mainly constituted of bacterial cells, later defined as the “gel layer” [33,34], whereas the effect of suspended solids was negligible. According to Wisniewski and Gransmick [35], the dissolved solids represent the fraction that most contributes to membrane fouling, representing 52% of the total resistance to filtration. On the contrary, according to Defrance et al. [36], a primary role is exerted by TSS (65%), whilst only 5% of the total resistance is due to dissolved solids, and the remaining 30% is imputable to colloidal matter. Finally, Bouhabila et al. [37] stated that the main fraction of total resistance was related to the colloidal matter, which contributes 51%, while TSS and the dissolved solids account for the 24 and 25%, respectively. 

In most practical applications, it is well accepted that biomass concentrations inside the bioreactor should not exceed the range of 8–12 gTSS L^−1^ [1]. It is worth noting that specific conditions (e.g., biological shocks, sharp variations in the operational conditions, toxics on the biomass) can affect the deposition trend, promoting peculiar effects not found in other experiments, which have been conducted under similar conditions.

This topic is still more contradictory considering the main influencing factors in the presence of high strength industrial wastewater [3]. Choo and Lee [38] analyzed an MBR system under a submerged configuration (submerged membrane bioreactor, SMBR) for the treatment of wastewater from a distillery. Choo and Lee [38] highlighted that the contribution of the intrinsic membrane to the filtration resistance was equal to 0.5%, while 82.8% was imputable to the internal irreversible fouling due to colloids and molecular macrospecies (internal pores clogging), and 16.6% was related to external deposition. Hai et al. [39] reported the results of an experimental study focused on the treatment of textile wastewater, demonstrating that fouling development was mainly due to the formation of a cake layer constituted of highly hydrophobic compounds, such as fungi and starch. Badani et al. [40], in a study focused on textile wastewater treatment, stated that membrane fouling depends on the stress level imposed on the biological flocs. Lastly, according to Mutamim et al. [6], fouling of the SMBR treating alkaline industrial wastewater is mostly imputable to high TSS concentrations, characterized by high viscosity levels.

Recently, many authors have agreed that one of the most important fouling factors is represented by the composition of extracellular compounds excreted by bacteria [41] and their specific interaction with the membrane surface and internal pores [4,7,42,43,44].

Many uncertainties and controversial opinions still remain on the actual role played by other operational parameters affecting MBRs features, referring in particular to membrane service life [7,8,16,45,46,47], the sludge retention time (SRT), the hydraulic retention time (HRT), and emerging micro-pollutants. This uncertainty highlights the complexity of the phenomenon and the toughness of its proper definition. Nevertheless, a very important parameter shared by the different fouling mechanisms is surely represented by the average size of the membrane pores, since it affects the balance between internal and external deposition. In this context, the cake layer deposition represents a further key factor affecting membrane fouling and the consequent permeate flux reduction [48]. The "biological membrane" constituted by the cake layer deposited on the membrane surface can positively operate as a pre-filter [49,50] or can negatively affect total membrane fouling [23,28,51,52].

The experimental observation of different fouling mechanisms can be improved by a complementary analysis of the effects deriving from the periodic cleaning operations of membrane modules, needed for the proper management of MBR systems [53,54,55,56,57]. Indeed, some of the first review papers on membrane cleaning operations have focused their attention on the fouling phenomena and control [12,58,59,60]. More specifically, membrane cleaning can involve simple or complex operations, featuring mechanical actions assisted by the use of chemicals, eventually focused on specific targets [61,62,63,64]. In this light, the experimental studies conducted on membrane cleaning operations have always been characterized by an increase in knowledge of different fouling mechanisms.

Bearing in mind the above considerations, the aim of the present paper was to summarize the main results achieved in experimental studies conducted in the past for the assessment of fouling mechanisms in MBRs by the application of the resistance-in-series (RIS) model during membrane physical cleaning operations. In particular, the different approaches reported in the literature about the “total resistance decomposition” are here presented and discussed. The core of the present work was to highlight the different data interpretation, suggesting at the same time the usefulness the application of the RIS model based on physical cleaning. In contrast, the detailed analysis/discussion of each fouling mechanism would require different work and was out of the scope of the present paper.

## 2. Analysis of Fouling Mechanisms

The fouling investigation analyzed the permeability variation as well as the deposition of solids on the membrane surface during the operation and/or individual filtration test [65,66,67,68,69]. In particular, many authors have analyzed the permeability of the perm-selective barrier (superficial deposition and physical membrane) basing on the total resistance to filtration. According to this approach, the overall fouling can be expressed as the sum of the different contributions related to specific fouling mechanisms, according to the RIS model [69,70,71,72,73]. 

The total resistance to filtration can be expressed by Equation (1), derived from Darcy’s law [1,71].

(1) $$R_{tot}=\frac{TMP}{J\cdot \mu }$$

where *J* represents the permeate flux (m^3^ m^−2^ s^−1^), *TMP* is the transmembrane pressure (Pa), *m* is the permeate viscosity (Pa s) and *R_tot_* is the total resistance to filtration (m^−1^). The RIS model and the estimate of each resistance contribution will be discussed in a section below. Nevertheless, on the basis of Equation (1), it is worth noting that the *R_tot_* is a function of *TMP* and permeate flux *J*, stating that the permeate viscosity is almost constant and equal to that of water at 20 °C. Therefore, from an operational point of view, the analysis of fouling development can be conducted by keeping constant either *TMP* or *J*, while enabling the variation of the second [74,75,76,77].

Moreover, the fouling investigation can be conducted by analyzing either the “reversibility” of the cake layer deposited on the membrane surface [70,78,79,80] or the different deposition mechanisms [55,71,81,82].

In agreement with the former approach, some authors differentiate the membrane fouling as "reversible" and "irreversible", on the basis of its "persistence to removal" after a specific cleaning action [55,83,84]. Accordingly, reversible fouling is when the deposit is easily removable through washing operations; conversely, irreversible fouling is when the residual deposit persists after washing operations. Depending on the different cleaning strategies, as well as the peculiar management of membrane modules, the actual evaluation of reversible and irreversible fouling is not trivial (and strictly related to the specific context of application); still representing the main challenge. As an example, when operating hollow fiber modules, generally submerged into a mixed liquor, reversible resistance can be imputable to the superficial deposition periodically removed by the regular backwashing with permeate [52] and/or the air scouring effect due to air bubble sparging [85]. On the other hand, irreversible resistance is characterized by persistent fouling only removable with ad-hoc cleaning operations, either physical or chemical [83,86,87].

The former reports on this topic [87,88] differentiated reversible from irreversible fouling on the basis of chemical cleaning actions only. In contrast, at present, it is generally accepted that an intense physical cleaning action enables the removal of a portion of the superficially deposited cake not removable during ordinary operations. Therefore, a portion of the superficial deposit can be evaluated as irreversible fouling [56,57,89,90].

More recently, the "irremovable fouling" concept was introduced, defined as the fouling portion that strongly affects the membrane service life as suggested by several plant operators and researchers [1,79]. Drews [79] even proposed to change the irreversibility concept into the "irremovability" one, introducing a classification aimed at confirming and extending this concept to the different membrane typologies working under different operational conditions. In this light, it is possible to define the reversible fouling as the portion constantly removed by the air scouring effect or by the ordinary backwashing, if present. The irremovable fouling is the aliquot not removed by ordinary actions but only removable by ad-hoc cleaning operations; either physical, chemical or a combination [64]. Lastly, irreversible fouling represents non-recoverable fouling and therefore membrane "ageing". However, the latter contribution can only be quantified by means of chemical cleaning actions. Therefore, the results obtained according to the previous approach (reversible/irreversible) should be re-interpreted in the light of Drews [79] classification, since the two approaches are partially overlapping. In this context, the irregular cleaning actions (physical and/or chemical) play a key role in the quantification of the different fouling contributions, other than in the recovery of membrane permeability.

The deposition of particles characterized by an average size larger than the membrane pores, defined as cake deposition, will produce superficial fouling that is mainly reversible [71,89]. Only a small amount of cake deposition will produce irreversible fouling and this portion is usually neglected, depending on the operational conditions or the adopted membrane modules [75,76,86,89]. Indeed, the evaluation of the complete or partial reversibility of the resistance due to cake deposition mainly depends on the imposed operational conditions [52,91].

Conversely, the resistance to filtration caused by particles having an average size smaller than the membrane pores will cause partial or total pore clogging, according to the following mechanisms [87]—complete blocking and standard blocking. These fouling mechanisms, originally introduced for dead-end filtration [92] are usually comprised of the general mechanism of pore blocking [82,93]. The internal fouling, due to pore blocking, is mainly irreversible and its partial removal can be obtained only through intense chemical cleaning actions [1,78,82,94]. Some authors state that there is a small amount of fouling due to pore blocking that can be removed by ordinary backwashing, even if this aliquot is negligible for permeate fluxes lower than 50 L m^−2^ h^−1^ [89]. Finally, it is possible to recognize a fouling mechanism related to the adsorption of foulants on the membrane, producing similar effects to the ones discussed previously (intermediate blocking). This mechanism considers the superficial adsorption that results in the clogging of the membrane pores, either totally or partially, as a consequence of gel layer deposition (polarization concentration) or macromolecule adhesion on the membrane surface [95,96]. In particular, “gel formation” is an intermediate blocking, almost irreversible, derived from a high concentration of an extracellular polymeric substance (EPS), especially tightly bound ones (TB-ESP).

**Figure 1:**
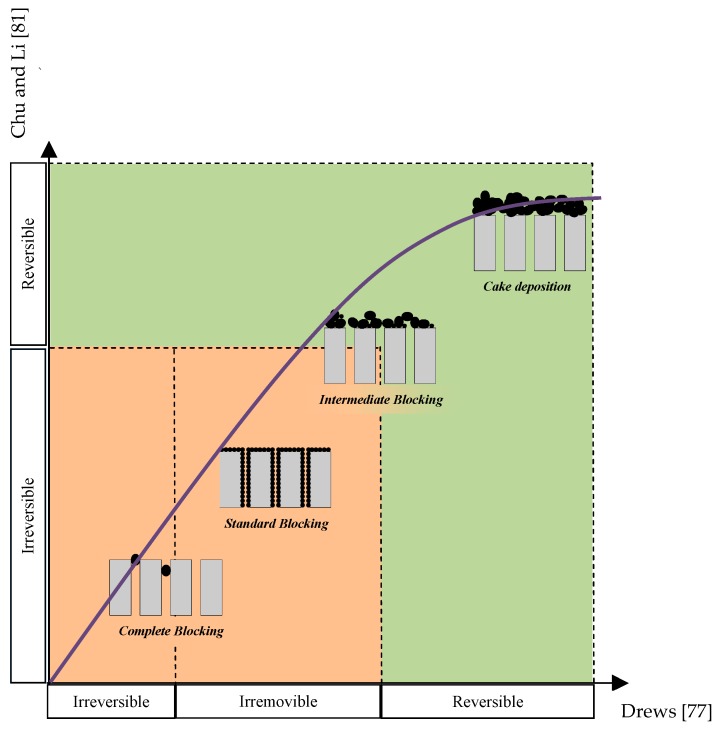
Reversibility of different fouling mechanisms.

Figure 1 schematically reports the four fouling mechanisms defined by Le-Clech et al. [59] compared with the classifications proposed by Chu and Li [83] (on the basis of the classical reversibility concept) and by Drews [79] (irreversible/irremovable deposition). From the analysis of Figure 1 it is worth noting that, while standard and complete blocking are mainly irreversible or irremovable, the cake deposition is almost reversible. In contrast, the intermediate blocking is the most complex mechanism and represents the origin of the main contradictions, in terms of reversibility and irreversibility; promoting discussions and debates within the scientific community.

The concepts reported in Figure 1 can be modelled by the approach proposed by Bowen et al. [97] and Hermia [98]; indeed, the aforementioned fouling mechanisms can be expressed in a general and consolidated form as:

(2) $$\frac{dJ}{dt}=-kJ{\left(A_{eff}\cdot j\right)}^{2n}$$

where *J* is the permeate flux (L m^−2^ h^−1^) at filtration time *t* (h); *k* is a constant dependent on the property of membrane; *A_eff_* is the effective membrane surface area (m^2^); *n* is the blocking index. When *n* equals 2, 1.5, 1 and 0 respectively, Equation (2) can correspondingly transform into the complete blocking model, standard blocking model, intermediate blocking model and cake filtration model, which are expressed by Equations (3)–(6) [99,100]:

(3) $$\frac{J}{J_{0}}=e^{-kt}=e^{-k_{a}j_{0}t}$$

(4) $$\frac{j}{j_{0}}={\left(1+2k_{S}A_{eff}\cdot J_{0}^{0.5}t\right)}^{-2}$$

(5) $$\frac{j}{j_{0}}={\left(1+2k_{S}A_{eff}\cdot J_{0}^{}t\right)}^{-1}={\left(1+k_{a}j_{0}t\right)}^{-1}$$

(6) $$\frac{j}{j_{0}}={\left(1+2kA_{eff}^{2}{}_{}\cdot J_{0}^{2}t\right)}^{-0.5}={\left(1+k_{C}R_{r}j_{0}t\right)}^{-0.5}$$

where *k_A_* is the blocked membrane surface per unit of the total volume permeated through the membrane (L m^−1^); *k_S_* is Hermia’s parameter, which describes the decrease in the cross-sectional area of the pores per unit of permeate volume (L m^−1^); *k_C_* is the area of cake per unit of permeate volume (L m^−1^); *R_r_* is the ratio of the cake resistance to the clean membrane resistance (dimensionless).

These models provide indications on the fouling mechanisms during filtration and they have been widely adopted in membrane fouling studies [101,102]. Other general aspects of fouling modelling will be discussed in the next paragraph.

## 3. Fouling Analysis with the RIS Model

In the technical literature, different approaches have been proposed with the aim to describe the manifold processes involved in membrane fouling, either external or internal. In particular, all of these studies have enabled the development of several mathematical models (mainly physical), including empirical models, fractal permeation models, sectional resistance models and RIS models, that are undoubtedly more complex compared to the conventional mass transfer and tangential filtration models [7,103]. 

The aim of the empirical models, mainly based on the hydrodynamic interpretation of the phenomenon, was to analyze the influence of the hydrodynamic conditions on the cross-flow velocity and superficial deposition [104]. These models are based essentially on the *TMP* measurement for the calculation of the membrane fouling rate, defined as the rate of resistance to filtration increase. According to this approach, the parameters describing the process are related to the cross-flow velocity imposed on the mixed liquor, aeration rate that regulates the wash-out of the material deposited on the membrane surface, mixed liquor viscosity, and the geometry of the reactor. All correlations have been developed through a careful analysis of the experimental hydrodynamic data. In particular, concerning the superficial fouling, the parameters of interest are the aeration rate, the permeate flux, and the concentration of suspended solids in the reactor. Moreover, the rheological features of the biofouling layer might affect significantly the evolution of the cake layer [105]. Briefly, membrane biofouling can be defined as a complex interaction process between a membrane and the mixed liquor components, involving bacteria, suspended solids, extracellular polymeric substances (EPS), bio-micromolecules, bio-macromolecules and colloidal matter into the cake layer [106]. The model equation shows explicitly the correlations between the several hydrodynamic parameters and the two most important components of MBR systems—the fouling rate and the cross-flow velocity. The latter is particularly crucial in defining the impact of the hydrodynamic conditions in terms of the formation and evolution of the cake layer on the membrane surface. Although this model is easy to use, it is too simple to clarify all of the complex phenomena involved in membrane fouling and, as a consequence, many operational parameters and specific working conditions are disregarded. Consequently, the empirical approach is not suitable for reproducing all the occurring phenomena.

The permeation model, based on a fractal analysis, has been developed to evaluate the permeability of the cake layer deposited on the membrane surface [107,108]. Since the cake microstructure is extremely complex and disordered, it is not possible to adopt traditional geometry concepts for its description. Therefore, the fractal permeation approach can be usefully applied, since it allows for the characterization of irregularly shaped objects through the description of average values, similitude or similar properties. As an example, Meng and co-workers [107] introduced a model for the evaluation of the superficial fractal size of the cake pores, basing the mathematical equation on the dimensional characteristics of typically sized pores as well as the cake layer (total area, pore edges, and sum of the area occupied by the characteristic pores). Such a model is more precise than the models previously developed by other authors and is based on fractal theory [108]. Indeed, Meng and co-workers [107] provided a rigorous procedure for the physical evaluation of the fractal size of the cake layer, including the use of an image analyzer for evaluating the area of every single pore. The permeability model was derived by an ad-hoc modification of the Hagen-Poiseulle equation in order to consider the flow velocity through the pores [109]. Its usefulness relies on the fact that the model holds a few parameters, is easy to determine, and does not require a significant computational burden. However, the model has been only indirectly validated and further developments are necessary to verify its applicability and the correlation between permeation factors (including pore size) and the effect on the cake layer resistance. Moreover, this approach does not illustrate how operational parameters and conditions could affect the cake resistance since it only provides the effects based exclusively on the fractal analysis.

The resistance study by means of sectional analysis has been mainly applied in order to quantify the total resistance starting from a detailed study of cake layer formation in SMBR systems [3,87,103]. Indeed, the cake layer formation is very irregular, especially in SMBR where the shear stress caused by aeration is unevenly distributed. Li and Wang [110], for instance, adopted this approach by ideally dividing the membrane surface into homogeneous filtration sections and calculating the total resistance for each section separately (subjected to a different hydrodynamic action depending on its position). The total resistance is computed as the sum of the different fouling mechanism contributions, analyzing each time the local flux and the sludge amount that is persistently deposited on the membrane surface. In particular, the superficial deposition is based on attachment and detachment related to the shear forces imposed by operational conditions such as permeate flux, mixed liquor concentration, particle size, and shear distribution related to tangential stress. The sectional resistance model was developed considering that a partial analytical approach has the aim of describing the fouling phenomenon in SMBR systems. This model enables the assessment of cake deposition/removal during both filtration and regular backwashing, quantifying the deposition over time [111]. However, the comparison between the measured and simulated *TMP* values highlights that the model is able to describe the overall fouling trend, whilst it is not suitable when the aim is to achieve a detailed description of the fouling deposition mechanisms. 

The resistance-in-series (RIS) model, despite some limitations, is likely the most complete and applied model, since it is easy to use and directly correlated to the phenomena under study [112]. However, it is worth noting that a recent study highlighted that the application of the RIS model has been criticized because the resistances might be not additive; fouling depends on the investigated conditions (e.g., sludge or supernatant filtering) [113].

The bibliographic review of the state-of-the-art, which was briefly discussed in the previous section, underlined that the correlation between membrane fouling and local parameters is quantitatively computable through the relationship between operational conditions (cross-flow velocity, MLSS concentration) and a peculiar parameter—the permeability or the resistance to filtration of the filtering system. Indeed, these parameters are mutually correlated through the permeate viscosity, through an inverse relationship—as the resistance increases, the system permeability decreases and vice-versa [87].

In the fouling analysis, as already pointed out, the hydraulic resistances are usually considered for the mathematical quantification of the fouling level. In particular, the filtering system (physical membrane plus internal and external fouling) is characterized by different resistance contributions describing the total resistance of the system. Usually, the approach adopted to describe the global effect is represented by the quantification of the total resistance as the sum of different resistances in series, each related to a specific fouling mechanism. According to Equation (1) (Section 2), the total resistance is mathematically defined by the permeate viscosity (*µ*) and the ratio between *TMP* and *J* [103]. Virtually, the "mechanistic filtration models", similarly to the RIS approach, are based on Darcy’s law as a theoretical starting point for the model equations. These equations directly relate the membrane flux (generally imposed by plant operators) to the measured *TMP* values—the permeate viscosity can be set as a constant or expressed as a function of temperature [7]. Darcy’s law (Equation (1)) enables the calculation of the total resistance as a combination of the original membrane resistance, *R_m_*, and the resistances deriving from the different fouling mechanisms [53,114]. Therefore, by applying the RIS model, the total resistance of the fouled membrane can be quantified as the sum of the intrinsic resistance of the clean membrane (*R_m_*) and the fouling resistance (*R_f_*). The latter, as aforementioned, can be split into different contributions due to the specific fouling mechanisms. The former approach, mostly adopted at the beginning, expresses the fouling resistance (*R_f_*) as the sum of the superficial deposition (cake deposition), *R_c_*, and the internal fouling (or pore blocking), R_PB_ [66,115].

(7) $$R_{tot}=\;R_{m}+\;R_{f}$$

(8) $$R_{tot}=\;R_{m}+\;R_{C}+\;R_{PB}$$

Nevertheless, due to the high number of foulant agents responsible for different fouling mechanisms, coupled to the contradictions that still exist in the technical literature, many authors have introduced different approaches for the "total resistance decomposition" classifications [7]. As an example, some authors have ascribed R_f_ entirely to pore blocking (*R_f_* = *R_PB_*), since it is assumed as the only resistance that actually alters the permeability of the physical membrane. In this light, the total resistance, reported in Equation (7), does not consider the term R_c_; that is computed separately [83,116]:

(9) $$R_{tot}=\;R_{m}+\;R_{C}+\;R_{f}$$

From a mathematical point of view, there is no evidence of differences between the two approaches. Indeed, according to Equation (9), *R_f_* is coincident with *R_PB_* and the sum of the three contributions corresponds to the *R_tot_* reported in Equation (8). However, a substantial and logical difference arises in the two classifications. Indeed, while Equation (8) considers the total resistance of the entire filtering system, that is the physical membrane plus the cake layer, Equation (9) expresses the alteration of membrane permeability in terms of reversibility by considering *R_f_* as the irreversible mechanism, whereas the contribution due to the cake layer (*R_c_*) is considered completely reversible. The two authors consider the same symbol (*R_f_*) to define a different effect despite the final result being the same in terms of total resistance. In the former approach (Equation (8)), both the cake and the pore blocking resistances can be characterized by a reversible and irreversible component, unless otherwise specified by the authors [117].

Therefore, it is possible to justify the existence of several resistances as reported in different experiments, where each contribution describes a specific fouling mechanism or a particular factor influencing the system. As an example, in the resistance model proposed by Li and Wang [110], based on the sectional analysis approach, the total resistance is computed as the sum of four contributions:

(10) $$R_{tot}=\;R_{m}+\;R_{p}+\;R_{sf}+\;R_{SC}$$

where *R_p_* is the resistance due to pore fouling; *R_sf_* is the resistance due to the "dynamic" deposition of a biological film onto membrane surface; *R_sc_* is the "persistent" fraction; while *R_m_* has been previously defined. A similar approach has been proposed by Chu and Li [83], with the only difference being that the *R_sc_* is the persistent contribution of the cake layer (and therefore not limited only to the biological film), whereas the biofilm resistance was totally included in *R_sf_* (dynamic or persistent).

Other authors [70,71,86] have computed separately the adsorption process, included in the term *R_ad_*, the concentration-polarization (*R_cp_*) and the membrane fouling (*R_f_*). Therefore, the total resistance to filtration will equal the sum of these contributions and the intrinsic membrane resistance *R_m_* according to Equation (11).

(11) $$R_{tot}=\;R_{m}+\;R_{ad}+\;R_{cp}+\;R_{f}$$

In particular, Busch et al. [71] concluded that the *R_cp_* is negligible, while the term *R_ad_* can be included within *R_f_* (also including the *R_c_* and *R_PB_* contributions) and the biofilm resistance (due to internal biofouling) is defined as *R_b_*. On the other hand, Choi et al. [70,86] characterized the reversibility of deposition, internal or external, by further dividing the term R_f_ into an irreversible portion (*R_if_*) and a reversible one (*R_rf_*). This is not necessarily coincident with the cake layer or pore blocking resistance.

Other authors have somehow exacerbated the concept of resistance in order to quantify a specific phenomenon or to highlight the effect of a class of fouling agents. For instance, Wisniewski and Grasmick [35] introduced resistances related to fouling caused by the sub-colloidal/colloidal fraction (*R_co_*) by the settleable fraction (*R_p_*) and by the soluble fraction (*R_s_*). The aim of such a classification was to characterize the filtration trials conducted with different solutions in order to assess the individual effect of each granulometric class. 

In Table 1, some of the most redundant resistances presented in the technical literature are reported. Despite many of the reported resistances have the same definition, they are identified with a different nomenclature due to the specific approach used. Furthermore, in some cases, the same nomenclature has been adopted to describe different fouling mechanisms. 

In this highly dispersive context, many authors proposed simplified classifications for the different resistances affecting membrane permeability from both a theoretical and/or practical point of view. Among the first approaches, the one proposed by Jiang et al. [89] was quite simple and considered the total resistance split in a series of resistances—each composed by a reversible and an irreversible portion. Subsequently, the resistance derived from the different mechanisms were listed in two different groups:

- “True/main resistances”, referred to the intrinsic membrane resistance (*R_m_*), cake resistance (*R_c_*) and blocking resistance (*R_p_*); the latter in particular is associated to internal fouling due to biofouling (on which the first approach was based) as well as to the inclusion within the pores of recalcitrant and inorganic compounds (as originally defined by Jiang et al. [89]);
- “estimated resistances”, such as the resistance of the fouled membrane due to irreversible fouling (*R_bw_*), represented by the sum of *R_m_* and the irreversible resistance (due to pore blocking, *R_irb_*, and/or cake, *R_irc_*), the resistance due to the reversible clogging of pores (*R_reb_*) and the reversible resistance of the cake (*R_rec_*).

**Figure 2:**
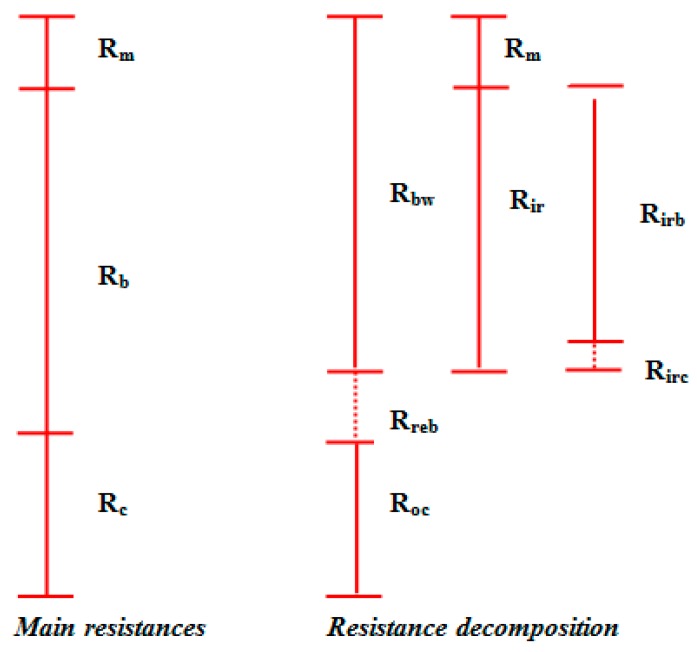
Example of different resistance decomposition (adapted from Jiang et al. [89]).

Figure 2 reports a schematic description based on a qualitative correlation among the resistances, which have been reported, according to the classification proposed by Jiang et al. [89].

The classification proposed by Jiang et al. [89], based on a "typical" filtration cycle, highlights the actual difference between the “main” resistances (*R_m_*, *R_b_*, *R_c_*) and the estimated characteristic resistances that, depending on the cleaning procedure adopted (irregular cleaning operation or ordinary backwashing), can include a reversible or irreversible fraction. The total resistance decomposition into separate portions is derived from the application of the RIS model (that enables a shift from the total resistance value to the specific resistances corresponding to the evaluated/evaluable fouling mechanisms). In this light, the fouling features should be a priori defined in order to properly assess the single terms of the equation (specific resistances); each defining a specific condition or process. Therefore, the repetition of the same contribution is avoided in the sum of the different resistances [7].

A direct and simple estimate of the resistances characterizing membrane fouling is usually conducted by measuring the variations of *J* or *TMP* during specific filtration tests [120,121]. Both Jiang et al. [89] and Di Bella et al. [75] evaluated the different resistances during the whole filtration/backwashing cycle through the measurement of the *TMP* variation at a constant permeate flux. 

In detail, according to the approach of Jiang and co-workers [89], the resistance *R_bw_* can be assessed from the initial *TMP* value immediately after the regular backwashing and represents the sum of the membrane resistance (evaluated in clean water under the same flux) and the irreversible fouling resistance *R_ir_*. The resistance *R_reb_*, only for flux values higher than 50 L m^−2^ h^−1^, is evaluated as the difference between the *TMP* at the end of the initial sharp increase (if any) and the initial *TMP* value. The resistances *R_rec_* and *R_co_* are computed through the subsequent gradual increase of *TMP*, as the difference between the *TMP* at the end of the filtration cycle and the *TMP* value at the end of the initial sharp increase. Finally, *R_irc_* is evaluated by the cake accumulation during the transition period, by comparing the variation of the filtration/backwashing flux and the cross-flow velocity (CFV) values, whereas the *R_irb_* is simply obtained as the difference between the *R_tot_* and the above terms, where *R_tot_* is evaluated from the final *TMP* value.

**Figure 3:**
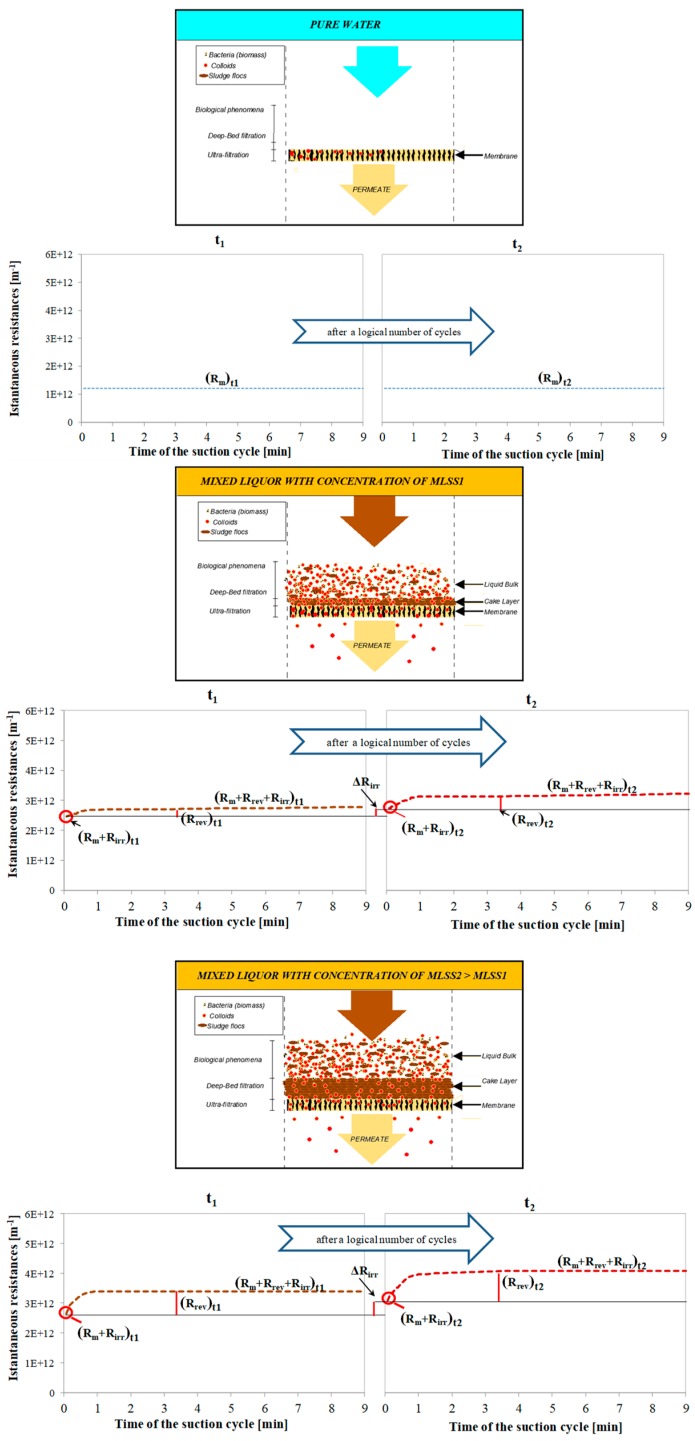
Evaluation of the fouling mechanism during a "typical filtration cycle" according to Di Bella et al. [55] (where: *t_1_* = time of observation related to the cycle 1; *t_2_* = time of observation related to the cycle 2 after a logical number of cycles; MLSS1 = concentration of suspended solids in the bioreactor at *t_1_*; MLSS2 = concentration of suspended solids in the bioreactor at *t_2_*).

The approach adopted by Di Bella et al. [55,75] is similar but it uses a different symbology. In particular, the authors assessed the resistances related to the different deposition phenomena/mechanisms basing on the analysis of different subsequent filtration cycles, with an approach similar to that proposed by Jiang et al. [89] through the instantaneous measurement of the resistance during the filtration cycles. The schematic calculation layout is shown in Figure 3. The analysis of the so-called "interest cycle" can be used to compare different filtration cycles over the time, after the accumulation of a considerable amount of *R_irr_*, which is evaluable after a "representative" number of filtration cycles, as defined by the authors, depending on the trend of the *TMP* profile. In this context, it is worth noting that the latter relies on many factors among which are the different compressibility of the cake layer and TSS concentration, almost at the beginning of the filtration cycle [52,122].

Meng et al. [64] adopted a different approach to characterize the terms reported in Equation (8), mainly based on flux variations. The resistances are evaluated using a three-step procedure: (1) the *R_m_* is measured by calculating the flux, under a constant *TMP* value, in clean water; (2) the *R_tot_* is evaluated on the basis of the final flux value after a microfiltration cycle of activated sludge; (3) at the end of the filtration cycle, the membrane is washed with a constant water flow, while scrubbing the surface with a sponge.

After this procedure, the membrane is placed again within the reactor (with activated sludge) and subjected to a filtration cycle with a resistance measurement; the hypothesis is that the latter value constitutes the sum of *R_m_* and *R_PB_*, from which it is possible to derive the term *R_PB_* by simply subtracting the value obtained at step 1. Finally, the *R_c_* is calculated from the *R_tot_* value obtained in step 2.

This approach represents one of the first protocols used for the quantification of the superficial cake deposition using “"physical cleaning”. It is important to stress that the approach based on simple physical cleaning can be applied only for the evaluation of the resistance due to superficial deposition. Indeed, analysis of the internal fouling is extremely complex, in terms of both feasibility and understanding and requires innovative and complex analytical techniques (SEM images, membrane autopsy) whose results are still a source of debate and contradiction within the scientific community [123,124].

## 4. Features of the Superficial Deposition and Role of Physical Cleaning

At present, the morphological study of the cake layer structure represents a challenging topic, with the aim of improving the knowledge level on the fouling mechanisms. Indeed, the filtration of complex fluids can produce an interaction between macromolecules and suspended particles that might affect the development rate of membrane fouling. In particular, the dynamics of adsorption/deposition phenomena significantly affect the filtration process since the specific development of fouling can cause a different decrease in the filtering system permeability (cake layer, or dynamic membrane, plus physical membrane). Moreover, the physical-chemical features of the superficial deposition, referring in particular to its “irreversibility”, are strictly related to the permeate flux as well as the mixed liquor composition. High or low flux values can differently affect the initial deposition of particles and the direct adsorption of macromolecules onto the membrane surface [50,125,126,127]. Generally, the “cake deposition” can be defined as the accumulation of particles on the external membrane surface. This process produces a further increase of the resistance to the permeate flux. Nevertheless, the cake layer can be characterized by different deposition typologies (solutes, particles and chemically active/non-active colloids). The different alternation among these layers strongly influences the cake layer role toward the effects and the features of fouling (cake filtration). The active foulants can go first to the membrane, interacting directly with the membrane surface and pores and forming a heterogeneous structure [77,128,129,130].

On the other hand, the formation of the first layer of suspended and inert particles on the membrane surface might reduce direct contact between the “active” foulants and the surface. In this case, the cake layer operates as a pre-filter, protecting the membrane from materials with a high fouling propensity [18,131,132].

**Figure 4:**
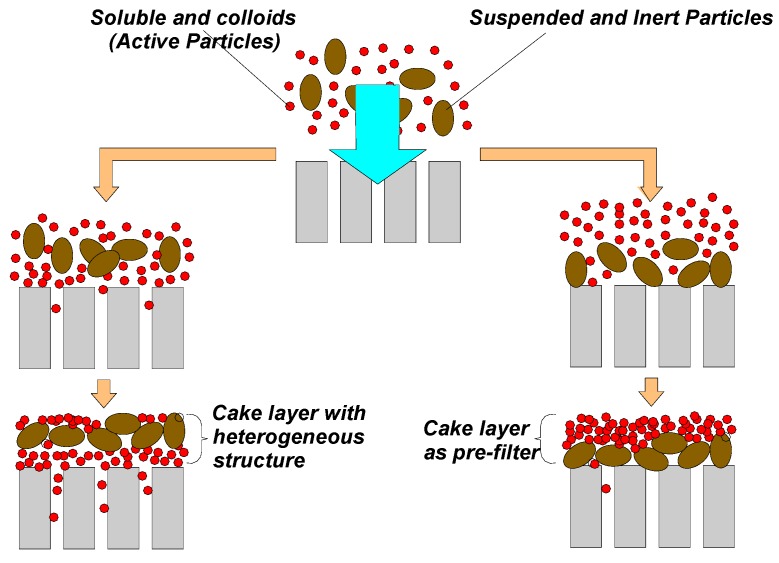
Different development of cake layer formation.

Figure 4 reports two different types of cake layer development that determine two morphologically distinct deposits, referring to both permeability and reversibility [122,128,129,131,132,133].

Direct deposition of colloidal and soluble molecules onto the membrane surface will induce the formation of a layered structure characterized by a mixture of suspended particles and macromolecules. On one hand, this fact will promote the adhesion of the suspended particles while, on the other hand, it will negatively affect the internal irreversible clogging as the smallest particles can be transported into the membrane pores due to the drag effect caused by the permeate flux [1,52].

Conversely, if the particles will deposit on the membrane surface first, it can enhance the formation of a layer acting as a pre-filter towards the macromolecules that could reach the membrane. However, this fact might not guarantee a higher membrane permeability as the superficial structure of the cake, composed by macromolecules and suspended particles, could determine a higher hydraulic resistance to filtration, despite the reversible features of the superficial deposition [50,52,73,75]. In general, high filtration fluxes and low a EPS concentration will promote the pre-filter. In contrast, a high EPS and poor flocs structure would promote a heterogeneous and worse cake layer.

The development of the superficial cake deposition, as well as the effect of suspended particles on fouling, is a complex phenomenon difficult to describe, since it depends on many factors, including the material and the morphology of the physical membrane [18,121]. However, many authors have tried to simplify the description of the deposition dynamics by introducing two fundamental groups of forces [13,110,111,132,134].

- Attachment/adhesion forces: mainly derived from permeate filtration and superficial electrostatic interaction;
- detachment forces: caused by turbulence induced by aeration, retro-fluxes (particle back-transport due to the cross-flow velocity) and permeate backwashing, if any.

Besides this dynamic stress, the "cake compressibility" should also be considered as it represents an indirect measure of its "density" level. In general, the external cake compressibility, either reversible or irreversible, relies on many factors, among which are the membrane module typology and the features of the mixed liquor [122,135,136]. In particular, the cake layer compression can modify its resistance ability and, as aforementioned, the typical trend of *TMP* profiles, especially after the preliminary "pre-compression" steps [52]. Many authors have investigated the development and the morphology of the cake layer and its effects on membrane fouling [137,138,139,140,141]. Some studies have highlighted that the superficial fouling resistance (and the negative effect of the cake compressibility level) can be reduced through the addition of suspended solids, like activated carbon or zeolites, or coagulant chemicals. Indeed, these compounds can promote the adsorption of specific organic molecules, such as polysaccharides and proteins, which constitute a primary source of macromolecule adhesion onto the membrane surface [22,142,143,144,145,146].

Similarly, the features of the cake layer deposited onto the membrane can be significantly affected by the "seeding" of a biofilm detached from suspended carriers, freely moving into the mixed liquor, in innovative MB-MBR (Moving Bed-MBR) or H-MBR (Hybrid-MBR) systems [23,76,147].

In this scenario, Panpanit and Visvanathan [148] analyzed the role of bentonite addition during the filtration of a mixture of emulsions and oils. The authors noticed that the addition of bentonite with a concentration higher than a specific threshold value drastically reduced membrane fouling, owing to the interaction between the emulsion and the suspended particles. Conversely, with a decrease in the bentonite dosage, a slow but constant flux decrease was observed, likely due to the deposition of a thicker cake on the membrane surface. In the latter case, the deposited layer was characterized by a composite nature or “mixed structure”, whereas higher bentonite dosages produced an ideal “pre-filter” (Figure 4).

In this context, it is also of interest to analyze the resistance behavior of the cake, after its deposition. Indeed, the cake layer provides additional resistance to filtration, depending on the typology of the particles and the consequent mutual interactions within the cake depth. In particular, the cake layer permeability is influenced by four major factors [56,76,91,147]:

1. salt content, whereby the permeability significantly decreases with an increase in the electrolytic concentration;
2. superficial potential of the particles that determines an intraparticle rejection that improves the cake layer permeability, under specific conditions;
3. size and morphology of the deposited particles that cause a minimum value of permeability, under specific conditions;
4. permeate flux that is inversely proportional to the cake layer compressibility.

Nevertheless, from a management point of view, the analysis and the evaluation of every single aspect afore listed do not exert a significant interest. Indeed, the filtration of a very complex mixed liquor such as the activated sludge, characterized by the presence of microorganisms and suspended particles, does not enable a reliable estimate of the deposition mechanisms. Moreover, the peculiar nature and morphology of microorganisms, changing over time, might modify the deposition phenomenon, improving or worsening the specific cake permeability from time to time [17,69,149,150,151,152,153].

Therefore, complete and careful comprehension of cake properties and their correlation with the physical membrane could be unnecessary. Indeed, during system operation, all experimental results on the microscopic scale reflect the macroscopic effects that influence the process. Furthermore, from an operational point of view, only a few microscopic phenomena can be directly affected—it is thus necessary to transfer this information into practical actions, by imposing specific operational conditions to the system, referring in particular to *TMP* and permeate flux values. These conditions should be considered during the ordinary operation of the system as well as during specific management operations, in order to better manage the filtration process according to the “guidelines” provided by experimental analysis. Nevertheless, the theoretical approach enables a better comprehension of reality and therefore the microscopic mechanisms affecting fouling. However, in this case, the experimental analysis would be mainly useful for scientific aims. 

In order to evaluate the cake layer resistance, representing in most cases the major contribution of membrane fouling for biological wastewater treatment [53,54,66,73,154], the cake layer deposited onto the membrane surface should be completely removed first [147]. Thereafter, by measuring the *TMP* and permeate flux values in clean water, it is possible to evaluate, according to Equation (1), the residual resistance due to the irreversible/irremovable fouling not removed (mainly internal pore blocking or residual intermediate blocking). Indeed, as previously pointed out, the study and the analysis of the effects of membrane cleaning operations have been undoubtedly useful as complementary information for a better comprehension of the different fouling mechanisms [58,155,156,157]. In particular, Shi et al. [27] state that “cleaning can be defined as a process whereby the material is relieved of a substance that is not an integral part of the material. A membrane cleaning should result in a membrane that is physically, chemically and biologically clean, and thus can provide adequate flux and separation”.

According to the aforementioned, many approaches exist for membrane cleaning. A general classification discriminates chemical cleaning from physical cleaning. The former is generally applied for irregular management operations and is aimed at removing the irreversible deposits, both internal and external, with aggressive “in place” cleaning operations (cleaning-in-place or CIP) or ex situ through an “out of place” operation (cleaning-out-of-place or COP) [62,158]. Obviously, since the effect of chemicals is quite aggressive, the optimal practice suggests reducing chemical cleaning operations to 1–2 per year [1].

On the other hand, physical cleaning is aimed at using “physical stress” (temperature, turbulence, mechanical action) in order to force the foulants deposited onto the membrane to leave the surface [27,157]. In the technical literature, this action is usually referred to as an "unconventional cleaning method". However, based on what has been afore-discussed, it appears as the most suitable procedure to remove the cake layer, reducing the chemical stress and, at the same time, providing useful and observable information for the quantitative evaluation and “nature” (reversible, irreversible, attached, thickened, "gluey") of the superficial deposited layer.

The superficial layer removal is usually performed by means of physical cleaning only. In this context, many methods have been proposed for the removal of the superficial deposition layer [159].

As an example, ultrasonication [160], water washing [161] or sponge scrubbing [21,66,162] have been proposed. It is obvious that the amount of the removed cake relies on many factors and can be significantly different depending on the specific adopted method. As a consequence, a standardized protocol does not yet exist. Indeed, these unofficial procedures reported in the technical literature have been accurately described and applied for a relative resistance analysis (having the aim to compare the results obtained in the same plants but in different moments) rather than an absolute one (comparison between plants characterized by different operational conditions) [52]. A first attempt to classify the different physical cleaning procedures has been proposed based on the different cleaning forces [26,157]; hydraulic or mechanic. In this context, it is possible to distinguish “back-flux” actions that oppose to the continuous *TMP* increase (as an example, the regular backwashing in hollow fiber membrane modules) from a huge turbulence increase or the application of "mechanical scouring" (for instance air scouring, sponge ball) [60,69,163,164,165,166,167,168]. It is not unusual that cleaning actions are coupled with membrane relaxation, in order to improve the cleaning efficiency [29,69,165,169,170].

In this wide scenario, the easiest management practices are those adopting simple mechanical and hydraulic actions according to the protocols reported in Table 2. At present, with the lack of a universally accepted procedure, physical cleaning has been judged useful only for a global analysis of fouling, considering a macroscopic approach that could be helpful in the management practice. Indeed, the cleaning efficiency is a function of the superficial cake features. The latter will influence the internal fouling and it is irreversible in nature. Nevertheless, in the last years, referring to pilot and bench scale plants, simple manual water rinsing has been applied several times for the limitation of fouling development, reducing the chemical cleaning frequency, if possible. Moreover, this approach has also been used to deepen the fouling analysis by means of total resistance decomposition, improving the comprehension of the involved mechanisms [56,76,171,172].

## 5. RIS Model Application during the “Manual Water Cleaning”

A major issue when using a non-standardized procedure for the definition of fouling mechanisms is related to the meaningfulness and reproducibility/repeatability of the results. Moreover, the scrubbing action might result “stressing” for the membrane. Indeed, some MBR operators have stated that “the sponge scrubbing is not scientific and is unrealistic for practical purposes” [178]. Conversely, the periodical air sparging/scouring is not reliable due to the instability and uneven distribution of the stress, strictly related to the mixed liquor viscosity [164,179,180]. Furthermore, the recurrence to ultrasonication has been limited due to the high energy consumption and the need for specialized operators [173,174,175,176,181]. In this context, only “water washing” could be easily applied during the normal operation of membrane modules. Nevertheless, due to a lack of standardized protocols, it cannot rise as an effective alternative to membrane chemical cleaning. This cleaning technique is quite interesting for experimental analyses aimed at characterizing membrane fouling due to cake deposition. Indeed, due to the simplicity of the approach, it was already adopted for the relative comparison of fouling under different operational conditions of several experimental pilot plants [55,56,76,182]. It is obvious that this cleaning procedure should be standardized for a more rigorous scientific use [27,52,183].

Recently, Di Bella et al. [57] reported a detailed analysis aimed at quantifying the reliability and repeatability of manual washing for the fouling mechanisms investigation. The aim of the study by Di Bella and co-authors [57] was to quantify the effect of membrane physical cleaning as well as to underline the consistency of the results of the RIS model application based on the "manual water washing" approach for the assessment of fouling mechanisms.

Physical cleaning has been used to define the characteristic resistances derived from the main fouling mechanisms. In detail, the adopted cleaning protocol was characterized by a series of different steps and actions reproduced almost identically by the operator, in all the investigated plants. The cleaning procedure consisted of an integrated combination of “manual water washing” and “shaker water washing” [57].

1. The permeate flux and transmembrane pressure were initially measured during normal operation, before the cleaning action. On the basis of these values, the total resistance to filtration can be evaluated, according to Equation (12):
  (12) $$R_{tot}{}_{,1}=\frac{TMP_{1}}{J_{1}\cdot \mu }$$
  where *J_1_* is the permeate flux of the fouled membrane (m^3^ m^−2^ s^−1^); *TMP_1_* is the transmembrane pressure in the same conditions (Pa); *µ* is the permeate viscosity (Pa s^−1^) that is almost equal to that of water at a working temperature.
2. Afterwards, the membrane was extracted from the bioreactor and physically cleaned through three operations in the following order. Firstly, membrane rinsing with tap water at 0.4–0.5 bar for 15 min, gently rubbing the membrane surface (or the membrane fibers in the case of hollow fiber modules). Thereafter, membrane cleaning was conducted in pure water by means of membrane shaking, either mechanical (for small membrane modules used in bench scale plants) or manual (for bigger modules used for pilot plant applications). Finally, 5 min of rinsing with ultrapure water (at low pressure, <0.2 bar).
3. Subsequently, the cleaned membrane was immersed in clean water and subjected to normal filtration (with the same operational flux and eventually with ordinary backwashing, if any) in order to measure the resistance to filtration in clean water, according to Equation (13):
  (13) $$R_{tot,cw}=\frac{TMP_{cw}}{J_{cw}\cdot \mu }$$
  where *J_cw_* (m^3^ m^−2^ s^−1^) and *TMP_cw_* (Pa) are the permeate flux and the transmembrane pressure in clean water after physical cleaning; *µ* is the water viscosity (Pa s^−1^).
4. The cleaned membrane was then immersed in the bioreactor and subjected to normal operational conditions (with the same fluxes and eventually ordinary backwashing). It is worth noting that the mixed liquor was the same as that at the beginning. On the basis of J_2_ and TMP_2_, it was possible to evaluate the total resistance to filtration, according to Equation (14):
  (14) $$R_{tot,2}=\;\frac{TMP_{2}}{J_{2}\cdot \;\mu }\;$$
  where *J_2_* is the permeate flux during the filtration of the same mixed liquor as that of step 1, after membrane physical cleaning (m^3^ m^−2^ s^−1^), *TMP_2_* is the transmembrane pressure under the same conditions (Pa), whereas *µ* is the permeate viscosity (Pa s^−1^).

Therefore, the RIS model has been applied in its simplest way. In particular, the total resistance *R_tot_*, which is usually computed by means of Equation (1), in the cleaning day coincides with *R_tot,1_*, previously defined. The cake contribution, both reversible and irreversible, has been subsequently computed in agreement with the approach discussed by Mannina and Di Bella [73], by combining Equations (8) and (9), after cleaning, according to steps 2 and 3 of the aforementioned protocol. 

In this context, the total resistance can be expressed based on the specific deposition mechanisms:

(15) $$R_{tot,1}=\;R_{m}+\;R_{PB}+\;R_{C,rev}+\;R_{C,irr}$$

The resistances *R_tot,cw_* and *R_tot,2_* can be expressed according to Equations (16) and (17):

(16) $$R_{tot,cw}=\;R_{m}+\;R_{PB}$$

(17) $$R_{tot,2}=\;R_{m}+\;R_{PB}+\;R_{C,rev}$$

Finally, the proposed RIS model has been applied in the following way, through Equations (18)–(20):

(18) $$R_{PB}=\;R_{tot,cw}-\;R_{m}$$

(19) $$R_{C,irr}=\;R_{tot,1}-\;R_{tot,2}$$

(20) $$R_{C,rev}=\;R_{tot,2}-\;R_{tot,cw}$$

**Figure 5:**
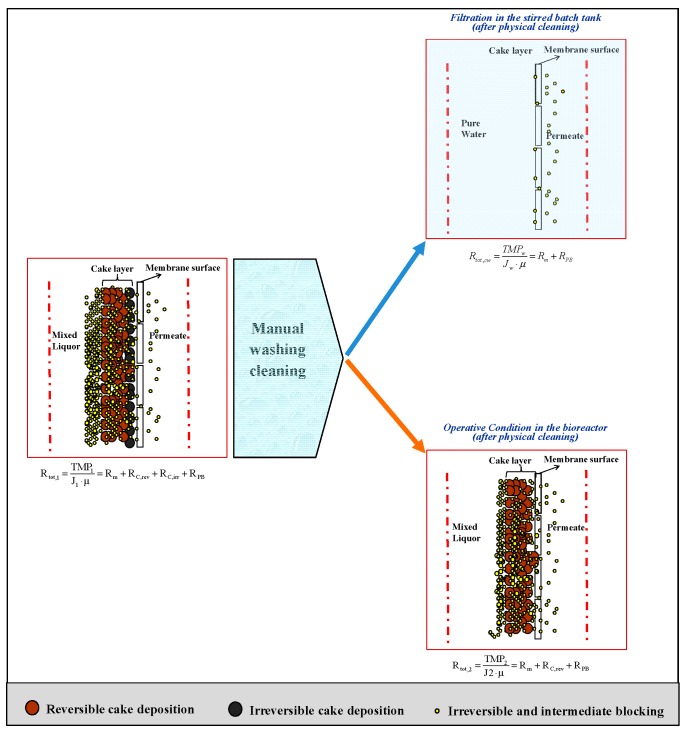
Logical scheme of RIS model based on the cake layer removal by means of physical cleaning.

**Figure 6:**
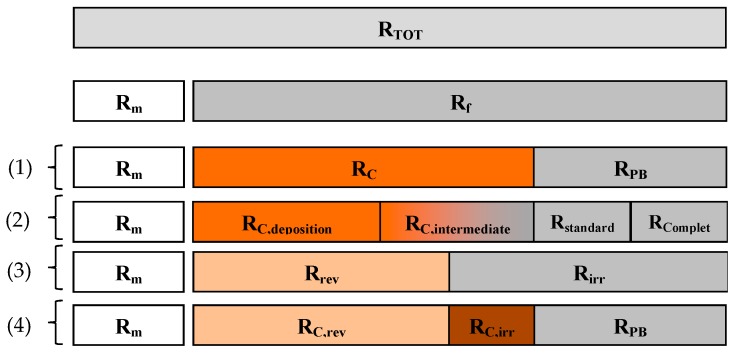
Resistances decomposition in different resistance-in-series (RIS) models applied; where: (1) is Chu and Li [83]; (2) is Le-Clech et al. [59]; (3) is Di Bella et al. [55]; (4) is Di Bella et al. [57].

Conceptually, the logical approach of the adopted RIS model is depicted in Figure 5. It is worth noting that the resistance decomposition described in Di Bella et al. [57] can be compared with previous classifications reported in the literature, as highlighted in Figure 6. It is important to highlight that chemical cleaning (following the advice of the manufacturers) should be required to properly quantify the irremovable contribution, according to the classification proposed by Drews [79]. Therefore, the RIS model should be re-arranged after the repetition of steps 3 and 4. However, the distinction between the irreversible and irremovable contribution was out of the scope of the present work. 

The application of a modified physical cleaning method reported by Di Bella et al. [57] demonstrates the applicability of the in-situ protocol for the analysis of in-depth fouling in order to elucidate the different fouling mechanisms through the application of the RIS model. The reproducibility of the method was excellent, for both bench and pilot scale plants. The results achieved with this simple method were in good agreement with the literature and were able to provide the analysis of fouling mechanisms enucleating the relative contributions of the different foulant contributors in terms of reversible and irreversible fouling. The method can be used to determine appropriate physical/chemical cleaning protocols for sustaining membrane permeability and potentially extending membrane service life, just based on the analysis of the role of the cake layer.

## 6. Future Perspectives for the Improvement of the “Manual Water Washing” Technique Adopted for Fouling Evaluation

The procedure described and discussed in the previous section represents, as endorsed by the obtained results, a useful tool for the analysis of fouling phenomena, referring in particular to scientific purposes. Indeed, what is discussed in the present work does not aim to introduce a new protocol but rather to highlight that the physical cleaning can be used to properly define the different contributions to be included in the RIS model, evaluating at the same time its consistency and repeatability. On the other hand, the application of manual water washing in full-scale plants, as previously discussed, needs further investigation in order to establish a standardized protocol, specific for the different typology of membrane modules. 

The main aims of physical cleaning might be summarized as follows:

- improve the overall efficiency, thus reducing the negative effects due to subsequent actions either chemical (aggressive action of chemicals) or physical (ultrasonic cavitation);
- reduce water, chemicals and energy consumption;
- minimize waste production and therefore negative impacts on the environment.

In this context, bearing in mind the complexity of fouling phenomena, other aspects should be considered to improve the efficiency of physical cleaning, such as pH and temperature. Although the inclusion of pH and temperature variation could increase the complexity of the protocol afore-discussed, its effect could be beneficial in terms of filtration recovery. In this sense, an improvement would regard the decrease and optimization of washing duration, rather than to the total amount of removed cake, that is already satisfactory, as afore-discussed. 

Moreover, the reported results refer to the same typology of membrane modules, realized with the same material (polyvinylidene difluoride (PVDF)) resulting in similar hydrophobic properties. However, despite the latter being the most frequently adopted in a submerged UF configuration [124,184], other materials with different properties (such as polymethyl methacrylate (PMMA), cellulose acetate (CA), polyacrylonitrile (PAN), polyethersulfone (PES), or polystyrene (PS) membranes) could determine different removal efficiencies and a consequential membrane fouling [11,185].

## 7. Conclusions

The present work focused on the role of the cake layer in the fouling analysis and its quantification through the application of the RIS model.

The superficial deposition onto a membrane plays a primary role in the overall fouling development. In particular, the bibliographic review highlighted that the cake layer contributes to the total resistance to filtration but it can have a beneficial effect as a foulant pre-filter.

In this context, irregular physical cleaning, which is a routine operation for the correct management of membrane fouling reducing the frequency of chemical cleanings, might extend the in-situ analysis of the fouling mechanism. The bibliographic review revealed how many authors have already employed the physical cleaning approach to quantify the contribution of different fouling mechanisms (cake deposition and pore blocking) in experimental plants. The analysis reported in the present work confirmed the usefulness of “manual water washing” as well as the consistency of the results obtained with an ad hoc RIS model application.

## Figures and Tables

**Table 1:** Specific resistances used in the total resistance decomposition.

Resistance	Mechanism Description	Main Classification	References
*R_ad_*	Resistance due to adsorption of particles matter onto the membrane	Intermediate blocking	Choi et al. [70,86] Busch et al. [71]
*R_b_*	Resistance due to blocking phenomenon	Standard blocking	Jiang et al. [89]
*R_c_*	Resistance due to cake deposition	Cake deposition	Lee et al. [115] Meng et al. [66] Wintgens et al. [116] Chu and Li [85] Ludwig et al. [118]
*R_bw_*	Resistance due to irreversible fouling of dissolved matter and colloids.	Intermediate blocking	Jiang et al. [89]
*R_irb_* or *R_b_*	Resistance due to internal irreversible fouling or internal blocking	Complete blocking	Jiang et al. [89] Broeckmann et al. [81]
*R_irc_*	Resistance due to superficial irreversible deposition	Intermediate blocking	Jiang et al. [89]
*R_reb_*	Resistance due to superficial reversible fouling or internal blocking	Intermediate blocking	Jiang et al. [89]
*R_rec_*	Resistance due to internal reversible fouling	Cake deposition	Jiang et al. [89]
*R_C,irr_*	Resistance due to irreversible cake deposition	Intermediate blocking	Jeison and van Lier [119] Di Bella et al. [55] Mannina and Di Bella. [73] Di Bella et al. [56] Di Trapani et al. [76]
*R_C,rev_*	Resistance due to reversible cake deposition	Cake deposition	Di Bella et al. [55] Mannina and Di Bella. [73] Di Bella et al. [56] Di Trapani et al. [76]
*R_co_*	Resistance do to internal deposition of colloids	Standard blocking	Wisniewski and Grasmick [35] Jiang et al. [89]
*R_cp_*	Resistance due to concentration by polarization	Intermediate blocking	Choi et al. [70,86] Busch et al. [71]
*R_f_*	Resistance of “Pore fouling”	Pore blocking	Lee et al. [115] Meng et al. [66] Wintgens et al. [116] Chu and Li [83] Ludwig et al. [118]
*R_p_*	Resistance due to irreversible pore blocking	Pore blocking	Bowen et al. [97] Chu and Li [83] Li and Wang [110] Broeckmann et al. [81]
*R_PB_*	Resistance due to pore blocking	Pore blocking	Lee et al. [115] Meng et al. [66] Diez et al. [52]
*R_sc_*	Resistance due to dynamic deposition of reversible biofouling	Intermediate blocking	Chu e Li [83] Li e Wang [110]
*R_sf_*	Resistance due to persistent deposition of irreversible biofouling	Standard blocking	Chu e Li [83] Li and Wang [110]
*R_rev_* or *R_rf_*	Resistance due to reversible fouling mechanism	Cake deposition intermediate blocking	Di Bella et al. [55] Mannina et al. [111] Diez et al. [52]
*R_irr_* or *R_if_*	Resistance due to irreversible fouling mechanism	Pore blocking intermediate blocking	Di Bella et al. [55] Mannina et al. [111] Diez et al. [52]

**Table 2:** Classification of different Physical cleaning strategies.

Denomination	Description	References
Water washing:	Can be performed in two different ways: with a shaker or manual. In the first case, “Shaker water washing”, the fouled membrane is placed in a tank containing a known volume of ultrapure water and shaken at a fixed speed (for a fixed time). In the case of “manual water washing” the membrane surface (or fiber) is rinsed under a stream of pressurized water and gently rub.	Chang et al. [161] Verberk and van Dijk [164] Liang et al. [167] Di Bella et al. [56] Cosenza et al. [171] Di Trapani et al. [76]
Ultrasonication	The membrane is placed in a container of a known volume containing ultrapure water and then subject to a typical ultrasound washing, similarly to the one used for washing the glassware in the laboratory. The contact time and the power may vary as a function of fouling.	Masselin et al. [173] Herbert et al. [160] Muthukumaran et al. [174] Li et al. [175] Maartens et al. [176]
Sponge scrubbing	The membrane is cleaned with a “sponge” until the surface of the membrane is apparently clean. This method is often used for a membrane with "flat panels"	Maartens et al. [176] Meng et al. [66] Psoch and Schiewer [177]
